# Analysis of Complete Chloroplast Genome Sequences Improves Phylogenetic Resolution in *Paris* (Melanthiaceae)

**DOI:** 10.3389/fpls.2016.01797

**Published:** 2016-11-29

**Authors:** Yuling Huang, Xiaojuan Li, Zhenyan Yang, Chengjin Yang, Junbo Yang, Yunheng Ji

**Affiliations:** ^1^Key Laboratory for Plant Diversity and Biogeography of East Asia, Kunming Institute of Botany, Chinese Academy of SciencesKunming, China; ^2^University of Chinese Academy of SciencesBeijing, China; ^3^Chinese Medicinal Resources Co. Ltd, Yunnan Baiyao GroupKunming, China; ^4^Germplasm Bank of Wild Species, Kunming Institute of Botany, Chinese Academy of SciencesKunming, China

**Keywords:** comparative genomics, phylogeny, chloroplast genome, *Paris*, *Daiswa*, Melanthiaceae

## Abstract

The genus *Paris* in the broad concept is an economically important group in the monocotyledonous family Melanthiaceae (tribe Parideae). The phylogeny of *Paris* was controversial in previous morphology-based classification and molecular phylogeny. Here, the complete cp genomes of eleven *Paris* taxa were sequenced, to better understand the evolutionary relationships among these plants and the mutation patterns in their chloroplast (cp) genomes. Comparative analyses indicated that the overall cp genome structure among the *Paris* taxa is quite similar. The triplication of *trn*I-CAU was found only in the cp genomes of *P. quadrifolia* and *P. verticillata*. Phylogenetic analyses based on the complete cp genomes did not resolve *Paris* as a monophyletic group, instead providing evidence supporting division of the twelve taxa into two segregate genera: *Paris sensu strict* and *Daiswa*. The sister relationship between *Daiswa* and *Trillium* was well supported. We recovered two fully supported lineages with divergent distribution in *Daiswa*; however, none of the previously recognized sections in *Daiswa* was resolved as monophyletic using plastome data, suggesting that the infrageneric relationships and biogeography of *Daiswa* species require further investigation. Ten highly divergent DNA regions, suitable for species identification, were detected among the 12 cp genomes. This study is the first successful attempt to provide well-supported evolutionary relationships in *Paris* based on phylogenomic analyses. The findings highlight the potential of the whole cp genomes for improving resolution in phylogeny as well as species identification in phylogenetically and taxonomically difficult plant genera.

## Introduction

The genus *Paris* in the wide sense (hereafter indicated by *Paris*), belongs to the tribe Parideae in the monocotyledonous family Melanthiaceae (Angiosperm Phylogeny Group, 2016), which comprises approximately 24 perennial herbaceous species, distributed throughout Europe and East Asia, with the majority of species (19/24) occurring in China (Li, 1998; Ji et al., 2006). *Paris* is well known in China for its medicinal qualities. The species with a thick rhizome (“medicinal *Paris*”) has been used as medicinal herb for more than 2000 years in China (Li, 1986), owing to its analgesic, hemostatic, anti-tumor, and anti-inflammatory activities (Long et al., 2003; He et al., 2006; Li et al., 2015). To date, more than 40 commercial drugs and health products have been developed in China using the rhizomes of “medicinal *Paris*” as raw materials (Li et al., 2015).

The classification of *Paris* is very complicated because of the plasticity of its morphological characteristics, and it has been subject to numerous critical revisions since the establishment. Hara (1969); Mitchell (1987, 1988), and Li (1998) recognized it as a single genus, whereas Takhtajan (1983) divided it into three genera: *Paris sensu strict* (*s.s*), *Daiswa*, and *Kinugasa*. The molecular phylogeny of *Paris* based on either single or multiple-locus DNA sequence data (e.g., *rbc*L, *mat*K, *trn*L/*trn*F, *psb*A/*trn*H and ITS) has remained controversial in recent investigations. The monophyly of *Paris* was justified by the studies of Osaloo and Kawano (1999) and Ji et al. (2006); however, analyses by Farmer and Schilling (2002) supported the taxonomical treatment of Takhtajan (1983). Despite recent insights into the evolutionary relationships within this plant group, a fully resolved and well-supported phylogeny remains elusive. It is, therefore, necessary to seek further evidence to reconstruct the phylogeny and to test the various classifications.

In addition, most *Paris* species have abundant intraspecific variations in morphology and chemical composition (Li, 1998; Ji et al., 2006; Wang et al., 2015). Inaccurate identification of these species could confound their effective exploration, conservation, and domestication. Moreover, at the species level, nearly all reported chloroplast (cp) DNA sequences (*rbc*L, *mat*K, *trn*L/*trn*F, and *psb*A/*trn*H) exhibit inadequate genetic variation (Osaloo and Kawano, 1999; Ji et al., 2006), to allow reliable discrimination of these species.

As complete cp genome sequences can offer valuable information for the reconstruction of complex evolutionary relationships in plants, they have been widely used for plant phylogenetic analyses and species identification in recent years (Jansen et al., 2007; Moore et al., 2007, 2010; Parks et al., 2009; Nock et al., 2011; Yang et al., 2013; Ruhsam et al., 2015). In the current study, we sequenced the complete cp genomes of eleven *Paris* taxa and compared these with the previously reported cp genome of *P. verticillata* (Do et al., 2014). The sampling covered almost half of species recognized by the updated classification (Li, 1998; Ji et al., 2006), and we carried out a comprehensive analysis of cp genomes in this phylogenetically and taxonomically difficult plant group. The primary objectives of the current study were: (1) to investigate the global cp genome structure of *Paris* species; (2) to test the previous classifications of *Paris* using complete cp genome sequences; and (3) to screen for sequence divergence hotspot regions among the twelve cp genomes as potential DNA barcodes for species identification.

## Materials and Methods

### Taxon Sampling, Sequencing, and Genome Assembly

Eleven taxa of *Paris* cultivated in the greenhouse in Kunming Institute of Botany, Chinese Academy of Sciences were sampled. Total genomic DNA was extracted from approximately 100 mg of clean, fresh leaves using the CTAB method (Doyle, 1987). Complete chloroplast genomes were amplified using Takara PrimeSTAR GXL DNA polymerase (Takara, Dalian, Liaoning, China) and nine universal pairs of primers and protocols developed by Yang et al. (2014). Purified PCR products were mixed and then digested into 200–500 base pairs (bp) fragments, and paired-end libraries were prepared according to the manufacturer’s manual (Illumina, San Diego, CA, USA). The libraries were sequenced using the Illumina Hiseq 2000 sequencing platform at BGI (Shenzhen, Guangdong, China).

Raw reads were filtered using NGSQC Toolkit (Patel and Jain, 2012), with the cut-off value for percentage of read length = 80, cut-off value for PHRED quality score = 30. High-quality reads were assembled into contigs using CLC Genomics Workbench v8.0 (CLC BIO, Aarhus, Denmark) with a minimum length of 1,000 bp. Next, all the contigs were aligned to the reference cp genome of *Paris verticillata* (KJ433485; Do et al., 2014), and aligned contigs were ordered according to the reference cp genome. Based on the reference cp genome, Contigs were reassembled and extended to obtain a complete cp genome sequence in Geneious 7.0 (Kearse et al., 2012), using the algorithm MUMmer. The validated complete cp genome sequences were deposited in GenBank (Supplementary Table S1).

### Genome Annotation and Comparison

Complete cp genomes were annotated using the Dual OrganellarGenome Annotator (DOGMA) database (Wyman et al., 2004). Start and stop codons and intron/exon boundaries were checked manually. Identified tRNA genes were verified using tRNAscan-SE 1.21 (Schattner et al., 2005) with the default parameters. The cp genome maps were drawn by the software OrganellarGenomeDRAW (Lohse et al., 2007). Comparison of the sequence divergence among the twelve cp genomes was performed using the mVISTA tool (Frazer et al., 2004) with the default parameters, and *P. verticillata* was set as a reference. To identify the mutations among 12 cp genomes, single nucleotide polymorphisms (SNPs) were identified using the tools embedded in Geneious 7.0 (Kearse et al., 2012), with the option setting as “Only Find SNPs.” Then, the variant frequency of SNPs in the protein coding and non-coding regions was calculated manually to detect the divergence hotspots across *Paris* cp genomes.

### Phylogenetic Analyses

The 12 completed *Paris* cp genomes were included in the analysis, of which 11 were newly generated in the current study (Supplementary Table S1). To reconstruct the phylogeny of *Paris*, eight species outside of *Paris* were included in the ingroup, representing all five tribes (Heloniadeae, Chionographideae, Xerophylleae, Melanthieae, and Parideae) recognized in the family Melanthiaceae (Angiosperm Phylogeny Group, 2016). The complete cp genomes from *Smilax china*, *Fritillaria cirrhosa*, and *Luzuriaga radicans* were used to root the tree. The published complete cp genomes were downloaded from the NCBI GenBank database (Supplementary Table S1).

Phylogenetic analyses were carried out by maximum likelihood analysis (ML) and Bayesian inference (BI). The ML analyses were performed using RAxML-HPC BlackBox version 8.1.24 (Stamatakis et al., 2008; Miller et al., 2010). The best-fitting substitution model was selected using ModelTest (Posada, 2008) and branch support was computed with 1,000 bootstrap replicates. The BI analyses were performed using MrBayes 3.2 (Ronquist and Huelsenbeck, 2003). Four Markov chains, starting with a random tree, were run simultaneously for one million generations, sampling trees every 2,000 generations. Trees from the first 250,000 generations were regarded as “burn in” and discarded, with posterior probability values determined from the remaining trees.

## Results

### Chloroplast Genome Features

**FIGURE 1:**
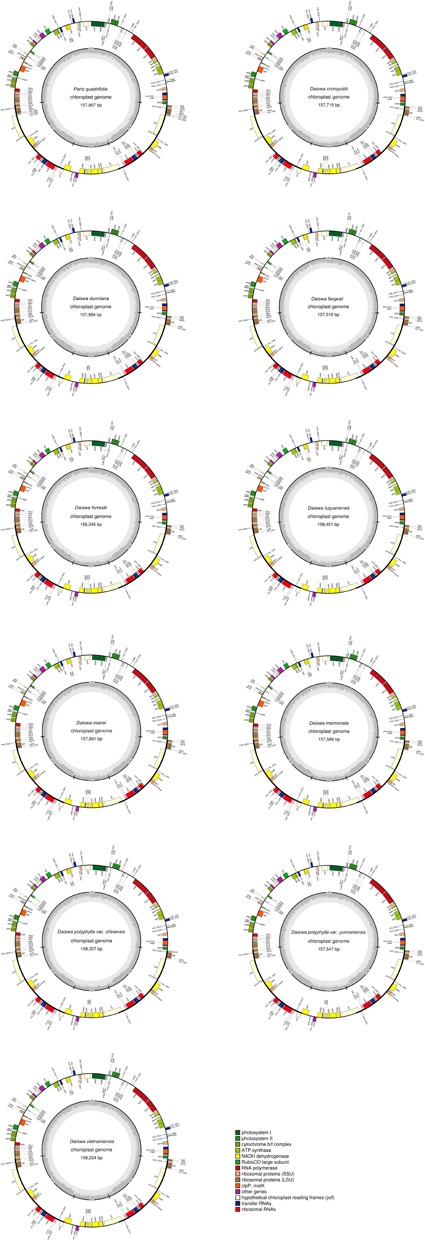
**Map of the 11 *Paris* chloroplast genomes newly generated in the current study**. Genes shown outside the circle are transcribed clockwise and those inside are transcribed counterclockwise. The dark gray area in the inner circle indicates the CG content of the chloroplast genome.

The twelve *Paris* complete cp genomes ranged from 157,379 to 158,451base paris (bp). All the cp genomes possessed the typical quadripartite structure of angiosperms, consisting of a pair of inverted repeated regions (IRs: 27,329–28,373 bp) separated by a large single-copy region (LSC: 82,726–85,187 bp) and a small single-copy region (SSC: 17,907–18,671 bp) (**Figure 1**; **Table 1**). All the 12 cp genomes possessed 115 unique genes arranged in the same order, including 81 protein-coding, 30 tRNA, and 4 rRNA genes. Of these, twelve protein-coding genes and six tRNAs contained at least one intron (**Table 2**).

**Table 1:** The comparison of the 12 *Paris* chloroplast genomes.

Taxa	Genome size (bp)	GC content (%)	LSC (bp)	SSC (bp)	IR (bp)	IR/SSC junction	IR/LSC junction
*D. marmorata*	157566	37.3	84221	18301	27522	*ycf*1	*rps*3
*D. forrestii*	158345	37.3	84396	18671	27639	*ycf*1	*rps*3
*D. polyphylla* var. *yunnanensis*	157547	37.3	84224	18319	27502	*ycf*1	*rps*3
*D. luquanensis*	158451	37.3	84408	18403	27820	*ycf*1	*rps*3
*D. marei*	157891	37.3	84420	18361	27555	*ycf*1	*rps*3
*D. vietnamensis*	158224	37.2	84794	18360	27535	*ycf*1	*rps*3
*D. fargesii*	157518	37.3	84549	18311	27329	*ycf*1	*rps*3
*D. cronquistii*	157710	37.3	84502	18316	27446	*ycf*1	*rps*3
*D. polyphylla* var. *chinensis*	158307	37.2	85187	18175	27473	*ycf*1	*rps*19
*D. dunniana*	157984	37.2	84482	18364	27569	*ycf*1	*rps*3
*P. verticillata*	157379	37.6	82726	17907	28373	*ycf*1	*rps*3
*P. quadrifolia*	157907	37.7	83772	18287	27924	*ycf*1	*rps*3

**Table 2:** List of genes encoded by 12 *Paris* chloroplast genomes.

Category of genes	Group of gene	Name of gene
Self-replication	Ribosomal RNA genes	*rrn*4.5*, rrn*5*, rrn*16*, rrn*23
	Transfer RNA genes	*trn*A_UGC*^∗^, trn*C_GCA*, trn*D_GUC*, trn*E_UUC*, trn*F_GAA*, trnf*M_CAU*, trn*G_GCC*, trn*G_UCC*^∗^, trn*H_GUG*, trn*I_CAU, *trn*I_GAU*^∗^, trn*K_UUU*^∗^, trn*L_CAA*, trn*L_UAA*^∗^, trn*L_UAG*, trn*M_CAU*, trn*N_GUU*, trn*P_UGG*, trn*Q_UUG*, trn*R_ACG*, trn*R_UCU*, trn*S_GCU*, trn*S_GGA*, trn*S_UGA*, trn*T_GGU*, trn*T_UGU*, trn*V_GAC*, trn*V_UAC*^∗^, trn*W_CCA*, trn*Y_GUA
	Ribosomal protein (small subunit)	*rps*2*, rps*3*, rps*4*, rps*7*, rps*8*, rps*11*, rps*12, *rps*12*^∗^, rps*14*, rps*15*, rps*16*^∗^, rps*18*, rps*19
	Ribosomal protein (large subunit)	*rpl*2*^∗^, rpl*14*, rpl*16*^∗^, rpl*20*, rpl*22*, rpl*23*, rpl*32*, rpl*33*, rpl*36
	RNA polymerase	*rpo*A*, rpo*B*, rpo*C1*^∗^, rpo*C2
	Translational initiation factor	*inf*A
Genes for photosynthesis	Subunits of photosystem I	*psa*A*, psa*B*, psa*C*, psa*I*, psa*J*, ycf*3*^∗∗^, ycf*4
	Subunits of photosystem II	*psb*A*, psb*B*, psb*C*, psb*D*, psb*E*, psb*F*, psb*H*, psb*I*, psb*J*, psb*K*, psb*L*, psb*M*, psb*N*, psb*T*, psb*Z
	Subunits of cytochrome	*pet*A*, pet*B^∗^*, pet*D^∗^*, pet*G*, pet*L*, pet*N
	Subunits of ATP synthase	*atp*A*, atp*B*, atp*E*, atp*F*^∗^, atp*H*, atp*I
	Large subunit of Rubisco	*rbc*L
	Subunits of NADH dehydrogenase	*ndh*A^∗^*, ndh*B^∗^*, ndh*C*, ndh*D*, ndh*E*, ndh*F*, ndh*G*, ndh*H*, ndh*I*, ndh*J*, ndh*K
Other genes	Maturase	*mat*K
	Envelope membrane protein	*cem*A*^#^*
	Subunit of acetyl-CoA	*acc*D
	Synthesis gene	*ccs*A
	ATP-dependent protease	*clp*P*^∗∗^*
	Component of TIC complex	*ycf*1
Genes of unknown function	Conserved open reading frames	*ycf*2*, ycf*15*^#^*

*^∗^With one intron; ^∗∗^With two introns; **^#^**Pseudogene.*

**FIGURE 2:**
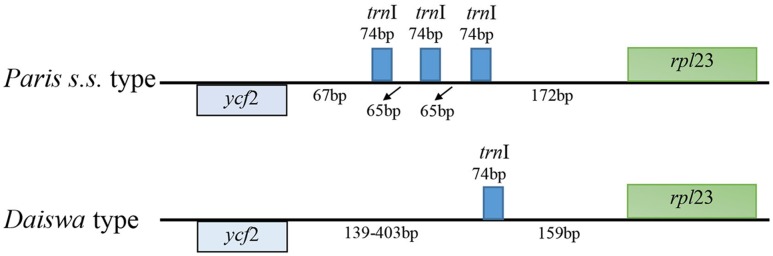
**The two types of *trn*I-CAU gene duplication detected in *Paris* taxa**.

The *Paris* cp genomes exhibited significant IR expansion. Expansion of IR regions into *rps3* at the IR/LSC boundaries was detected in all taxa, except for *Daiswa polyphylla* var. *chinensis*, where the IR regions expanded into *rps19* (**Table 1**). Expansion of the IR region into the *ycf1* pseudo-gene at IR/SSC junction regions occurred in all *Paris* taxa, leading to an overlap between the *ycf1* pseudo-gene and *ndh*F (**Table 1**). The length of the intergenic spacer between *rpl23* and *ycf2* was highly variable among the twelve cp genomes. Two patterns of variation (designated as *Paris s.s.* and *Daiswa* types) based on the number of copies of *trn*I-CAU were observed (**Figure 2**). The *Paris s.s* type possessed three copies of *trn*I-CAU, and was present in *P. verticillata* and *P. quadrifolia*. The *Daiswa* type, including only a single copy of *trn*I-CAU, was identified in the remaining taxa.

### Phylogenomic Analyses

**FIGURE 3:**
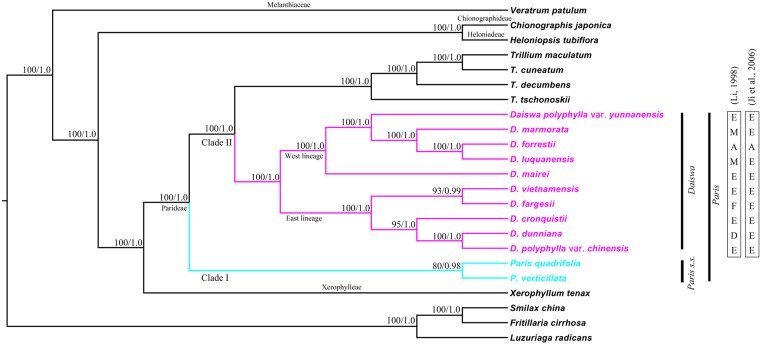
**Maximum likelihood (ML) phylogeny of Melanthiaceae based on complete chloroplast genomes inferred from 20 taxa representing all five tribes of the family**. Numbers indicate bootstrap values >80% from the ML analyses and posterior probabilities >0.90 from the Bayesian inference (BI) analyses. Section delimitations in the *Daiswa* species reported by Li (1998) and Ji et al. (2006) are shown on the right. A, sect. *Axiparis*; D, sect. *Dunnianae*; E, sect. *Euthyra*; F, sect. *Fargesianae*; M, sect. *Marmoratae*.

Phylogenetic relationships within the Melanthiaceae family were reconstructed by ML and BI analyses. The resulting ML and BI tree topologies were highly similar to one another. **Figure 3** illustrates the phylogeny generated by ML analysis, including two types of support values: ML bootstrap values (MLBS) and BI posterior probabilities (PP). Both analyses fully supported the monophyly of the tribe Parideae (*Trillium + Paris*) (MLBS = 100% and PP = 1.00). The basal divergence within the Parideae formed two major clades (I and II). Clade I (MLBS = 80% and PP = 0.98) comprised *P. quadrifolia* and *P. verticillata*, corresponding to the *Paris s.s* outlined by Takhtajan (1983). Clade II was resolved as two subclades (MLBS = 100%, PP = 1.00): *Trillium*, and another, consisting of species placed in the genus *Daiswa* by Takhtajan (1983). The phylogenetic relationships recovered by analysis of whole cp genome sequences did not support *Paris* as a monophyletic group.

The results of both ML and BI analyses provided significant evidence to support a sister relationship between *Trillium* and *Daiswa* (MLBS = 100% and PP = 1.00). We recovered two lineages (MLBS = 100% and PP = 1.0) in the *Daiswa* clade; one comprised taxa (*D. polyphylla* var. *chinensis*, *D. dunniana*, *D. cronquistii*, *D. fargesii*, and *D. vietnamensis*) distributed from eastern to central China and Vietnam (the east lineage); whereas the other comprised the species, *D. mairei*, *D. luquanensis*, *D. forrestii*, *D. marmorata*, and *D. polyphylla* var. *yunnanensis*, which are distributed from southwest China to the Himalayas (the west lineage). However, none of the sections in the *Daiswa* proposed by either Li (1998) or Ji et al. (2006) was resolved as monophyletic (**Figure 3**).

### Sequence Divergence Hotspot Regions

**FIGURE 4:**
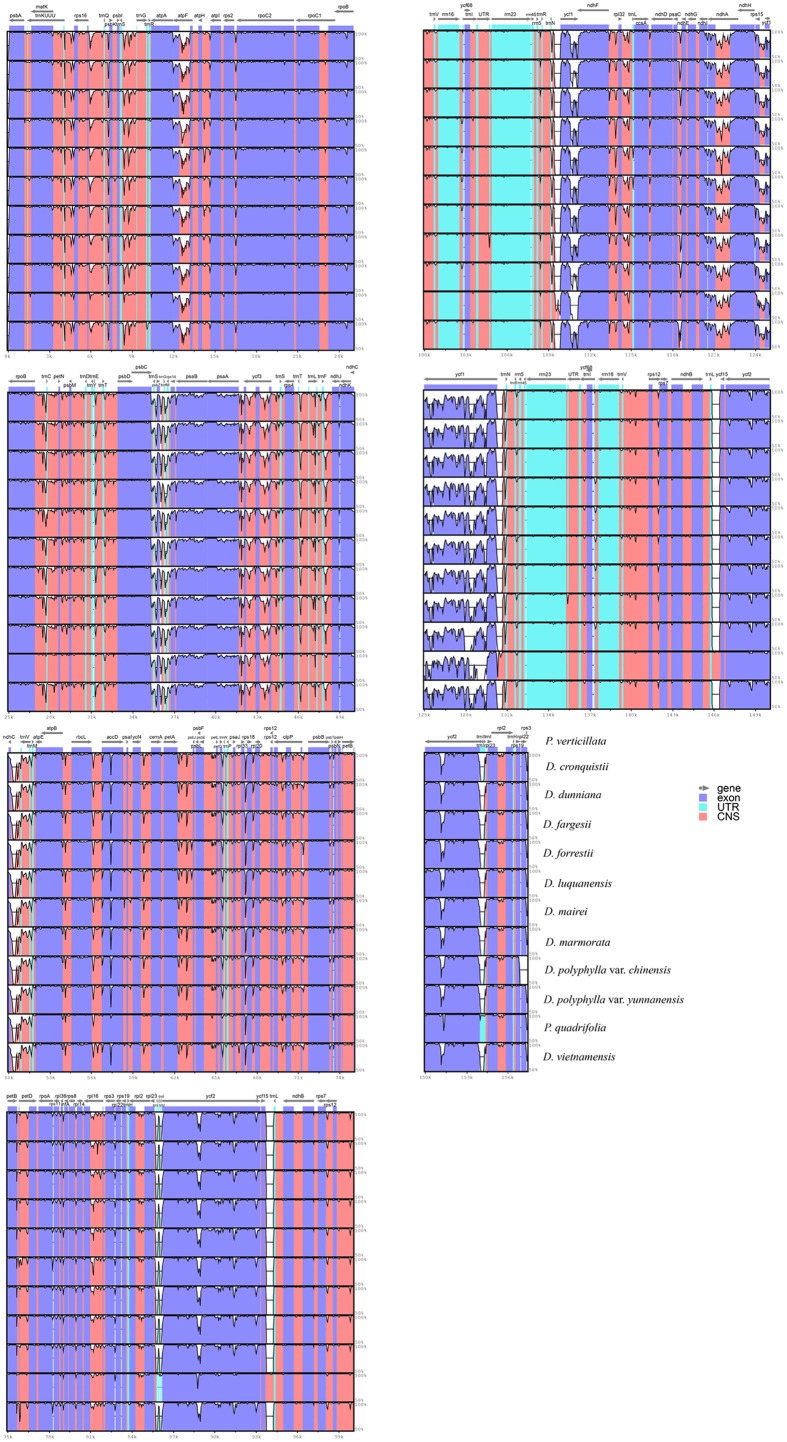
**Sequence identity plots for the 12 *Paris* taxa**.

Regions containing sequence divergence hotspots were identified by cp genome-wide comparative analyses (**Figure 4**). Single nucleotide polymorphisms (SNPs) are the most important marker for species identification (Kress et al., 2005). To identify DNA regions that could be suitable for discriminating *Paris* species, SNPs across the twelve complete cp genomes were comprehensively examined. We detected 2,748 SNPs (1.756%) among the cp genomes, in which protein-coding genes and non-coding regions (introns and spacers) exhibited divergence proportions of 1.655 and 2.033%, respectively (Supplementary Table S2). Among these divergence hotspot regions (Supplementary Tables S3 and S4), we screened 10 non-coding regions with potential to be useful loci for the molecular identification of *Paris* species, with lengths ranging from 200 to 1,500 bp and percentages of SNPs exceeding 3%. Primers for these plastid DNA markers are presented in **Table 3**.

**Table 3:** Potential DNA barcodes to identify *Paris s.s.* and *Daiswa* species.

Loci	Location	Primers	GC %	Tm (°C)
*ndh*C*/trn*V-UAC	LSC	F:ACAAAACTTTCTCGCTCGGT R:TTCTATGGACCAAGCAACCG	45.0 50.0	58.1 57.9
*trn*N-GUU*/ycf*1	IRB	F:CCGGAACTTCTTCGTAGTGG R:CCCCGAAGTGGCTCTATTTC	55.0 55.0	58.0 58.0
*rps*15*/ycf*1	SSC	F:CATCTGGTATACGCAAAAGCG R:ACCTATGCGTACATCTTTCGG	47.6 47.6	57.8 57.9
*rpl*33*/rps*18	LSC	F:AACAAAACGCGTGTTCGATC R:ATTTCGGCCGGATCTGAAAT	45.0 45.0	58.0 57.7
*ndh*A intron	SSC	F:ACCCATGTAATTCTGTCGGC R:GGGGAAGTACTGCTTGATCG	50.0 55.0	58.0 58.1
*atp*F intron	LSC	F:TTTGGCTCTCACGCTCAATT R:TCGCTTCGGCATTGGATAAA	45.0 45.0	58.1 58.0
*psb*Z*/trn*G-GCC	LSC	F:CCTCGATTCAAAAATGCCGT R:GCGAAAATATGATCCAGACGC	45.0 47.6	57.1 57.8
*psa*A*/ycf*3	LSC	F:ACAAAGAGACCTGCCAACAG R:TGCAACCGAGTCCTAGTGTA	50.0 50.0	58.0 58.1
*trn*V-UAC intron	LSC	F:ACCTTGACTTAGGTCTGCCT R:CAAATCGATGGCGGGTTCTA	50.0 50.0	58.0 58.1
*ccs*A*/ndh*D	SSC	F:GGTTCTCAAAAACTCTAGAGGC R:TTGCATTCTACAGCGAACGA	45.5 45.0	56.7 57.9

## Discussion

### Comparative Genomics

Our results revealed that the overall gene content and arrangement within the 12 *Paris* taxa are largely similar. The IR/LSC boundaries in *Paris* cp genomes (other than those of *D. polyphylla* var. *chinensis*) expand into *rps3*. This differs from the typical monocot genome structure, in which IR regions expand into *rps19* (Kim and Lee, 2004; Yang et al., 2013). Among other taxa in the family Melanthiaceae, IR expansion into *rps3* has been observed in *Chionographis japonica* (Chionographideae; Bodin et al., 2013) and *Xerophyllum tenax* (Xerophylleae; Do et al., unpublished data). *Veratrum patulum* (Melanthieae; Do et al., 2013); *Heloniopsis tubiflora* (Heloniadeae; Do et al., unpublished data); *Trillium tschonoskii*, *T. decumbens*, *T. cuneatum*, and *T. maculatum* (Parideae; Kim et al., 2016; Schilling et al., unpublished data; and Schilling et al., unpublished data; and Kim et al., 2016; respectively),exhibit the typical monocot genome structure at their IR/LSC junctions. This suggests that the expansion of the IR/LSC junctions into *rps3* may have occurred independently during the evolutionary history of the family Melanthiaceae, and may not provide relevant phylogenetic information.

Gene duplications in the cp genomes of higher plants have mainly been found in tRNA genes (Hipkins et al., 1995). Three copies of *trn*I-CAU, located between *rpl23* and *ycf 2*, were found in the cp genome of *P. verticillata* and *P. quadrifolia* in the current study; however, this feature was not identified in the remaining *Paris* taxa, or in previously examined monocot cp genomes (Do et al., 2014). The triplication of the *trn*I-CAU gene may be unique to *Paris* taxa, and could thus provide useful information contributing to the exploration of evolutionary relationships.

### Phylogenetic and Taxonomic Resolution

The utilization of too few DNA sequence may result in the incongruence between DNA regions, and can increase the phylogenetic errors (Rokas and Carroll, 2005; Philippe et al., 2011). Therefore, phylogenetic analysis of plant species using a small number of loci might be frequently insufficient to resolve evolutionary relationships, particularly at low taxonomic levels (Parks et al., 2009). The molecular differences in complete cp genome between plant species can offer promising evolutionary information (Jansen et al., 2007; Parks et al., 2009). As a result, the cp genomes sequencing could greatly improve the phylogenetic resolution at low taxonomic levels (Parks et al., 2009; Ruhsam et al., 2015; Williams et al., 2016). Nevertheless, using the complete cp genome to reconstruct evolutionary relationship in those phylogenetically and taxonomically difficult genera has been rarely investigated (Ruhsam et al., 2015; Williams et al., 2016).

The key interest in the current study is to resolve the previously phylogenetic controversies in *Paris* (Osaloo and Kawano, 1999; Farmer and Schilling, 2002; Ji et al., 2006) by using the complete cp genome sequences. Our phylogenomic analyses did not resolve *Paris* as a monophyletic group, and strongly supported its division into two monophyletic genera: *Paris* *s.s.* and *Daiswa* (**Figure 3**). This treatment is justified by both morphological and geographical evidence (**Table 4**). Species belonging to *Paris s.s.* have a long, slender rhizome, a round ovary, and seeds without sarcotesta or aril. In contrast, *Daiswa* species have a thick rhizome, an angular ovary, and seeds covered by juicy sarcotesta or aril (Li, 1998; Ji et al., 2006). In addition, *Paris* species are concentrated in temperate areas of Eurasia, whereas those belonging to *Daiswa* are distributed in subtropical and tropical areas of East Asia. It is notable that the triplication of *trn*I-CAU was observed only in *Paris s.s.*, and not in either *Daiswa* or other monocots (Do et al., 2014; Kim et al., 2016; the current study), which may provide further comparative genomic evidence to support this generic circumscription.

**Table 4:** Critical characters for *Daiswa*, *Paris* *s.s.* and *Trillium*.

Genus	Distribution	Rhizome	Leaves	Flower	Ovary	Seed	No. of *trn*I-CAU copy in cp genome
*Daiswa*	Subtropical and tropical areas of East Asia	Thick	A whorl of 4–15 net-veined leaves at stem apex	Solitary	Angular	With sarcotesta or aril	one
*Paris s.s.*	Temperate areas of Eurasia	Long and slender	A whorl of 4–15 net-veined leaves at stem apex	Solitary	Rounded	Without sarcotesta or aril	Triplication
*Trillium*	North America and East Asia	Thick	A whorl of 3 net-veined leaves at stem apex	Solitary	Angular	Without sarcotesta or aril	duplication


Our phylogenomic analyses also well resolved the inter-tribe relationships in the family Melanthiaceae and the inter-generic relationships within the tribe Parideae, with higher support than previous phylogenetic studies that used single or multiple locus DNA sequences data (Osaloo and Kawano, 1999; Farmer and Schilling, 2002; Ji et al., 2006; Kim et al., 2013). This result was consistent with previous findings (Attigala et al., 2016) in which a much higher of support to inter-generic relationships was observed in the cp genomic phylogeny within Arundinarieae tribe (Bambusoideae: Poaceae). The sister relationship between *Paris s.s.* and *Daiswa* + *Trillium* clade can be justified by the morphological synapomorphies of single whorl of net-veined leaves at stem apex and solitary flower (**Table 4**). In addition, plants of *Daiswa* and *Trillium* share a thick rhizome and an angular ovary (Ji et al., 2006), which are probably the synapomorphies grouping these two genera. Nevertheless, a question that remains unresolved by our study is the phylogenetic position of *Paris japonica* (or *Kinugasa japonica*). This species was placed into the monotypic genus *Kinugasa* by Takhtajan (1983). As we did not obtain a sample of this plant, the generic circumscription of *Kinugasa* and its relationships to the other Parideae genera will require further investigation.

Compared to previous molecular phylogeneitc analyses (Osaloo and Kawano, 1999; Ji et al., 2006; Farmer and Schilling, 2002), our results clearly indicated that all nodes within the *Daiswa* clade showing a MLBS > 90% and PP > 0.95 (**Figure 3**). Similar results have also been observed from the whole chloroplast genome analysis of *Pinus* species (Parks et al., 2009), *Araucaria* species (Ruhsam et al., 2015), and *Acasia* species (Williams et al., 2016). Nevertheless, relatively lower node support within *Paris s.s.* was observed (MLBS = 80%, PP = 0.98, **Figure 3**). Given that only two species were included in the analyses, this may result in a phylogeny that is more sensitive to homoplasy, and can thus decrease the phylogenetic resolution (Wiens, 2003). Therefore, a much larger taxon sample may provide a better resolution of the infra-generic relationships and species identification in the *Paris s.s.*, as previous studies indicated (Williams et al., 2016).

It is notable that none of the sections within *Daiswa* which were proposed by either Li (1998) or Ji et al. (2006) was resolved as monophyletic through our analyses of the complete cp genomes. This implies that the previous delimitation of the sections must be reassessed. We recovered two fully supported cladest within the *Daiswa*. Species within the two lineages have distinctive distribution patterns, with the east lineage being distributed from eastern and central China to Vietnam and the west lineage from southwestern China to the Himalayas. This implies that the extant *Daiswa* species that occurred between these two geographical regions could have experienced long-term vicariance. However, the sampling within *Daiswa* in this study may be too low to satisfactorily address this issue, and maternally inherited plastomes can only provide partial insight into evolutionary history (Triplett et al., 2014). The evolutionary relationships and biogeography of *Daiswa* species require further investigation, including increased sampling of species and infra-specific populations, and application of additional nuclear DNA markers.

### Potential DNA Barcodes

Because of the plasticity of the morphological characteristics among *Paris* species, its taxonomy remains problematic. The plastid loci, *rbc*L, *mat*K, and *psb*A/*trn*H, are recommended as universal DNA barcodes in plants (Hollingsworth et al., 2011); however, we found that the percentage of variation in *rbc*L and *mat*K were relatively low (1.046 and 0.773%, respectively) among *Paris* species (Supplementary Table S3). Due to the expansion of the IR into the LSC region, three protein-coding genes (*rps*3, *rps*19, and *rpl*22) were inserted into the *psb*A/*trn*H-GUG spacer of *Paris* species (**Figure 1**). This cp genome rearrangement could account for the significantly increased length of the *psb*A/*trn*H region among these taxa (∼1,200 bp), in which the divergence proportion is unexpectedly low (Ji et al., 2006; Yang et al., 2011). As a result, these three universal plastid DNA barcodes have extremely low power to identify either *Paris* *s.s.* or *Daiswa* species. Thus, the novel DNA barcodes are urgently needed.

The mutation events in the genome were not random but clustered as “hotspots,” which created the highly variable regions throughout the complete cp genomes (Shaw et al., 2007). These sequence divergence hotspot regions could provide adequate genetic information for species identification, and can be used to develop novel DNA barcodes (Parks et al., 2009; Yang et al., 2013). We propose ten plastid DNA regions harboring a high proportion of SNPs (**Table 3**), which are potentially useful for species identification in *Paris* *s.s* and *Daiswa*. The *ndh*A intron and *atp*F intron have been widely used for phylogenetic studies (Shaw et al., 2007). The rest eight loci harboring highly genetic variations are newly identified in the current study. Our further research will investigate whether these DNA sequences could serve as reliable and effective DNA barcodes for species from *Paris s.s.* and *Daswa*, the two medicinally important genera. We also encourage researchers working on other plant groups to use these loci developed in this study for phylogenetic reconstruction and species identification.

## Conclusion

This study is the first attempt to reconstruct phylogeny in *Paris* with the taxon sample covering 50% known species which represents a wide phylogenetic diversity in this medicinally important plant group. The overall cp genome structure across these plants is highly conserved. The phylogenomic analyses provided the most strongly supported estimate of evolutionary relationships among *Paris* taxa, which supports the division of these taxa into two segregate genera: *Paris* *s.s* and *Daiswa*. Our study resolved the debates in phylogeny and classification of *Paris*. Ten rapidly evolving regions were identified across the cp genomes that could serve as potential DNA barcodes for species identification in *Paris* *s.s* and *Daiswa*. The findings justify that the whole cp genome sequencing can offer plenty genetic information for resolving evolution and species identification in those phylogenetically and taxonomically difficult plant genera.

## Author Contributions

YJ designed the research; YH, XL, CY, ZY, and JY collected and analyzed the data; YJ, YH, and JY prepared the manuscript.

## Conflict of Interest Statement

The authors declare that the research was conducted in the absence of any commercial or financial relationships that could be construed as a potential conflict of interest.
