# Supplementary material for: Analysis of Complete Chloroplast Genome Sequences Improves Phylogenetic Resolution in Paris (Melanthiaceae)
Source: Front Plant Sci. 2016 Nov 29;7:1797. doi: 10.3389/fpls.2016.01797 (PMC5126724; doi:10.3389/fpls.2016.01797)
Supplement: Supplementary file 1 [file Table_1.DOCX]

**Table S1. GenBank accession numbers for the complete chloroplast genomes of *Paris* and related genera*.***

| **Taxa** | **Accession number** | **References** |
| --- | --- | --- |
| *Chionographis japonica* | KF951065 | Bodin *et al.*, 2013 |
| *Fritillaria cirrhosa* | KF769143 | Li *et al.*, 2014 |
| *Heloniopsis tubiflora* | KM078036 | Do *et al.*, unpublished |
| *Luzuriaga radicans* | KM233640 | Kim *et al.*, 2014 |
| *Daiswa cronquisitii* | KX784041 | The current study |
| *Daiswa dunniana* | KX784042 | The current study |
| *Daiswa fargesii* | KX784043 | The current study |
| *Daiswa forrestii* | KX784044 | The current study |
| *Daiswa luquanensis* | KX784045 | The current study |
| *Daiswa mairei* | KX784046 | The current study |
| *Daiswa marmorata* | KX784047 | The current study |
| *Daiswa polyphylla* var. *chinensis* | KX784048 | The current study |
| *Daiswa polyphylla* var. *yunnanensis* | KX784049 | The current study |
| *Daiswa vietnamensis* | KX784050 | The current study |
| *Paris quadrifolia* | KX784051 | The current study |
| *Paris verticillata* | KJ433485 | Do *et al.*, 2014 |
| *Trillium cuneatum* | KR135077 | Schilling *et al.*, unpublished |
| *Trillium decumbens* | KR534612 | Schilling *et al*., unpublished |
| *Trillium maculatum* | KR780075 | Kim *et al.*, 2016 |
| *Trillium tschonoskii* | KR780076 | Kim *et al.*, 2016 |
| *Veratrum patulum* | KF437397 | Do *et al.*, 2013 |
| *Xerophyllum tenax* | KM078035 | Do *et al.*, unpublished |
