# Supplementary material for: Analysis of Complete Chloroplast Genome Sequences Improves Phylogenetic Resolution in Paris (Melanthiaceae)
Source: Front Plant Sci. 2016 Nov 29;7:1797. doi: 10.3389/fpls.2016.01797 (PMC5126724; doi:10.3389/fpls.2016.01797)
Supplement: Supplementary file 2 [file Table_2.DOCX]

**Table S2. Summary of SNPs found in the twelve *Paris* complete chloroplast genomes.**

| **Data type** | **Number of SNPs** | **Characters(bp)** | **Divergence proportion (%)** |
| --- | --- | --- | --- |
| Complete chloroplast genome | 2,748 | 156,522 | 1.756 |
| Protein-coding genes | 1,334 | 80,590 | 1.655 |
| Non-coding regions | 1,295 | 63,705 | 2.033 |
