# Supplementary material for: Analysis of Complete Chloroplast Genome Sequences Improves Phylogenetic Resolution in Paris (Melanthiaceae)
Source: Front Plant Sci. 2016 Nov 29;7:1797. doi: 10.3389/fpls.2016.01797 (PMC5126724; doi:10.3389/fpls.2016.01797)
Supplement: Supplementary file 3 [file Table_3.DOCX]

**Table S3. SNPs detected in protein-coding genes across the twelve *Paris* complete chloroplast genomes.**

| Protein-coding region | Characters  (bp) | SNP | Divergence proportion (%) | Location |
| --- | --- | --- | --- | --- |
| ycf1 | 5,577 | 490 | 0.087860857 | SSC/IRA |
| rps15 | 273 | 18 | 0.065934066 | SSC |
| ycf1 | 1,236 | 79 | 0.063915858 | IRB |
| atpF | 555 | 30 | 0.054054054 | LSC |
| ndhA | 1,092 | 55 | 0.050366300 | SSC |
| rps14 | 303 | 15 | 0.049504951 | LSC |
| psaB | 2,205 | 89 | 0.040362812 | LSC |
| ndhC | 363 | 14 | 0.038567493 | LSC |
| clpP | 591 | 18 | 0.030456853 | LSC |
| psaA | 2,253 | 65 | 0.028850422 | LSC |
| rps11 | 399 | 10 | 0.025062657 | LSC |
| ndhI | 543 | 12 | 0.022099448 | SSC |
| ycf3 | 513 | 10 | 0.019493177 | LSC |
| psbT | 108 | 2 | 0.018518519 | LSC |
| ndhF | 2,238 | 39 | 0.017426273 | IRB/SSC |
| psbZ | 189 | 3 | 0.015873016 | LSC |
| rpl20 | 354 | 5 | 0.014124294 | LSC |
| ndhD | 1,503 | 17 | 0.011310712 | SSC |
| rps3 | 657 | 7 | 0.010654490 | LSC/IRB |
| rpl23 | 282 | 3 | 0.010638298 | IRB |
| rpl23 | 282 | 3 | 0.010638298 | IRA |
| rbcL | 1,434 | 15 | 0.010460251 | LSC |
| rpoB | 3,213 | 31 | 0.009648304 | LSC |
| psbM | 105 | 1 | 0.009523810 | LSC |
| ycf2 | 7,089 | 64 | 0.009028072 | IRA |
| ycf2 | 7,089 | 64 | 0.009028072 | IRB |
| psaI | 111 | 1 | 0.009009009 | LSC |
| psbH | 222 | 2 | 0.009009009 | LSC |
| petG | 114 | 1 | 0.008771930 | LSC |
| psbF | 120 | 1 | 0.008333333 | LSC |
| psaC | 246 | 2 | 0.008130081 | SSC |
| matK | 1,554 | 12 | 0.007722008 | LSC |
| rpoC1 | 2,079 | 16 | 0.007696008 | LSC |
| psbN | 132 | 1 | 0.007575758 | LSC |
| rps8 | 399 | 3 | 0.007518797 | LSC |
| rps19 | 279 | 2 | 0.007168459 | IRB |
| rps2 | 711 | 5 | 0.007032349 | LSC |
| rpl32 | 150 | 1 | 0.006666667 | SSC |
| rps7 | 468 | 3 | 0.006410256 | IRB |
| rps7 | 468 | 3 | 0.006410256 | IRA |
| rpoC2 | 4,110 | 26 | 0.006326034 | LSC |
| ccsA | 966 | 6 | 0.006211180 | SSC |
| rpl2 | 822 | 5 | 0.006082725 | IRB |
| rpl2 | 825 | 5 | 0.006060606 | IRA |
| ndhH | 1,182 | 7 | 0.005922166 | SSC |
| rpoA | 1,023 | 6 | 0.005865103 | LSC |
| rpl14 | 369 | 2 | 0.005420054 | LSC |
| ycf4 | 555 | 3 | 0.005405405 | LSC |
| psbK | 186 | 1 | 0.005376344 | LSC |
| ndhK | 768 | 4 | 0.005208333 | LSC |
| infA | 201 | 1 | 0.004975124 | LSC |
| atpE | 405 | 2 | 0.004938272 | LSC |
| rpl16 | 411 | 2 | 0.004866180 | LSC |
| accD | 1,482 | 7 | 0.004723347 | LSC |
| cemA | 694 | 3 | 0.004322767 | LSC |
| petD | 483 | 2 | 0.004140787 | LSC |
| atpH | 246 | 1 | 0.004065041 | LSC |
| psbE | 252 | 1 | 0.003968254 | LSC |
| ndhG | 531 | 2 | 0.003766478 | SSC |
| rps4 | 606 | 2 | 0.003300330 | LSC |
| rps18 | 306 | 1 | 0.003267974 | LSC |
| petB | 648 | 2 | 0.003086420 | LSC |
| psbD | 1,062 | 3 | 0.002824859 | LSC |
| psbC | 1,422 | 4 | 0.002812940 | LSC |
| atpI | 744 | 2 | 0.002688172 | LSC |
| rps12 | 372 | 1 | 0.002688172 | IRA |
| rps12 | 372 | 1 | 0.002688172 | IRB |
| atpB | 1,497 | 4 | 0.002672011 | LSC |
| atpA | 1,524 | 4 | 0.002624672 | LSC |
| psbB | 1,527 | 4 | 0.002619515 | LSC |
| rpl22 | 384 | 1 | 0.002604167 | IRB |
| petA | 963 | 2 | 0.002076843 | LSC |
| ndhB | 1,533 | 2 | 0.001304631 | IRB |
| ndhB | 1,533 | 2 | 0.001304631 | IRA |
| psbA | 1,062 | 1 | 0.000941620 | LSC |
