# Supplementary material for: Analysis of Complete Chloroplast Genome Sequences Improves Phylogenetic Resolution in Paris (Melanthiaceae)
Source: Front Plant Sci. 2016 Nov 29;7:1797. doi: 10.3389/fpls.2016.01797 (PMC5126724; doi:10.3389/fpls.2016.01797)
Supplement: Supplementary file 4 [file Table_4.DOCX]

**Table S4. SNPs detected in non-coding regions across the twelve *Paris* chloroplast genomes.**

| Intron and spacer | Character(bp) | SNP | Divergence proportion | Location |
| --- | --- | --- | --- | --- |
| *trn*I-CAU2/trnI-CAU3 | 65 | 29 | 0.446153846 | IRA |
| *trn*I-CAU3/trnI-CAU2 | 65 | 27 | 0.415384615 | IRB |
| *ndh*C*/trn*V-UAC | 726 | 99 | 0.136363636 | LSC |
| *ycf*1/trnN-GUU | 668 | 90 | 0.134730539 | IRA |
| *trn*N-GUU/ycf1 | 668 | 89 | 0.133233533 | IRB |
| *ndh*I*/ndh*A | 88 | 11 | 0.125000000 | SSC |
| *psb*C*/trn*S-UGA | 139 | 16 | 0.115107914 | LSC |
| *rps*15*/ycf*1 | 407 | 42 | 0.103194103 | SSC |
| *trnf*M-CAU*/rps*14 | 168 | 14 | 0.083333333 | LSC |
| *ndh*H*/rps*15 | 100 | 8 | 0.080000000 | SSC |
| *trn*S-UGA*/psb*Z | 194 | 15 | 0.077319588 | LSC |
| *trn*I-CAU2*/trn*I-CAU1 | 65 | 5 | 0.076923077 | IRB |
| *rpl*33*/rps*18 | 224 | 16 | 0.071428571 | LSC |
| *ndh*A intron | 1,060 | 72 | 0.067924528 | SSC |
| *atp*F intron | 784 | 52 | 0.066326531 | LSC |
| *psb*Z*/trn*G-GCC | 287 | 19 | 0.066202091 | LSC |
| *trn*I-CAU1*/trn*I-CAU2 | 65 | 4 | 0.061538462 | IRA |
| *rps*14*/psa*B | 133 | 8 | 0.060150376 | LSC |
| *trn*G-GCC*/trnf*M-CAU | 184 | 11 | 0.059782609 | LSC |
| *psa*A*/ycf*3 | 404 | 24 | 0.059405941 | LSC |
| *trn*V-UAC*/trn*M-CAU | 162 | 9 | 0.055555556 | LSC |
| *trn*V-UAC intron | 602 | 32 | 0.053156146 | LSC |
| *ycf*3 intron | 1,646 | 83 | 0.050425273 | LSC |
| *rpl*23*/trn*I-CAU3 | 172 | 6 | 0.034883721 | IRB |
| *trn*I-CAU3*/rpl*23 | 172 | 6 | 0.034883721 | IRA |
| *ccs*A*/ndh*D | 234 | 8 | 0.034188034 | SSC |
| *trn*Y-GUA*/trn*E-UUC | 59 | 2 | 0.033898305 | LSC |
| *ndh*E*/ndh*G | 181 | 6 | 0.033149171 | SSC |
| *ycf*2/trnI-CAU1 | 67 | 2 | 0.029850746 | IRA |
| *ndh*F*/rpl*32 | 762 | 21 | 0.027559055 | SSC |
| *psb*I*/trn*S-GCU | 112 | 3 | 0.026785714 | LSC |
| *rpl*32*/trn*L-UAG | 829 | 21 | 0.025331725 | SSC |
| *rpl*36*/inf*A | 158 | 4 | 0.025316456 | LSC |
| *trn*W-CCA*/trn*P-UGG | 160 | 4 | 0.025000000 | LSC |
| *psa*C*/ndh*E | 324 | 8 | 0.024691358 | SSC |
| *trn*Q-UUG*/psb*K | 354 | 8 | 0.022598870 | LSC |
| *rpo*B*/trn*C-GCA | 841 | 18 | 0.021403092 | LSC |
| *atp*I*/rps*2 | 235 | 5 | 0.021276596 | LSC |
| *trn*L-UAA intron | 545 | 11 | 0.020183486 | LSC |
| *trn*S-GGA/*rps*4 | 308 | 6 | 0.019480519 | LSC |
| *trn*L-UAA/*trn*F-GAA | 375 | 7 | 0.018666667 | LSC |
| *trn*F-GAA/*ndh*J | 644 | 12 | 0.018633540 | LSC |
| *rps*8*/rpl*14 | 173 | 3 | 0.017341040 | LSC |
| *trn*S-GCU*/trn*G-UCC | 1,083 | 18 | 0.016620499 | LSC |
| *ndh*D*/psa*C | 122 | 2 | 0.016393443 | SSC |
| *rpl*2 intron | 672 | 11 | 0.016369048 | IRA |
| *rpl*2 intron | 675 | 11 | 0.016296296 | IRB |
| *rps*3*/rpl*22 | 63 | 1 | 0.015873016 | IRB |
| *rpl*14*/rpl*16 | 127 | 2 | 0.015748031 | LSC |
| *atp*B*/rbc*L | 700 | 11 | 0.015714286 | LSC |
| *mat*K*/rps*16 | 1,570 | 24 | 0.015286624 | LSC |
| *rps*18*/rpl*20 | 262 | 4 | 0.015267176 | LSC |
| *ndh*G*/ndh*I | 267 | 4 | 0.014981273 | SSC |
| *rps*11*/rpl*36 | 134 | 2 | 0.014925373 | LSC |
| *trn*I-CAU1*/ycf*2 | 67 | 1 | 0.014925373 | IRB |
| *rpo*A*/rps*11 | 68 | 1 | 0.014705882 | LSC |
| *acc*D*/psa*I | 273 | 4 | 0.014652015 | LSC |
| *trn*G-UCC*/trn*R-UCU | 144 | 2 | 0.013888889 | LSC |
| *trn*C-GCA*/pet*N | 828 | 11 | 0.013285024 | LSC |
| *psb*A*/mat*K | 534 | 7 | 0.013108614 | LSC |
| *atp*H*/atp*I | 618 | 8 | 0.012944984 | LSC |
| *psa*J*/rpl*33 | 465 | 6 | 0.012903226 | LSC |
| *clp*P intron | 1,408 | 18 | 0.012784091 | LSC |
| *rpl*16/*rps*3 | 160 | 2 | 0.012500000 | LSC |
| *trn*L-UAG/*ccs*A | 81 | 1 | 0.012345679 | SSC |
| *rps*2/*rpo*C2 | 248 | 3 | 0.012096774 | LSC |
| *ycf*3/*trn*S-GGA | 622 | 7 | 0.011254019 | LSC |
| *ndh*J/*ndh*K | 89 | 1 | 0.011235955 | LSC |
| *psb*B/*psb*T | 178 | 2 | 0.011235955 | LSC |
| *clp*P/*psb*B | 451 | 5 | 0.011086475 | LSC |
| *pet*B intron | 814 | 9 | 0.011056511 | LSC |
| *trn*T-GGU/*psb*D | 1,001 | 11 | 0.010989011 | LSC |
| *pet*L/*pet*G | 184 | 2 | 0.010869565 | LSC |
| *ycf*4/*cem*A | 739 | 8 | 0.010825440 | LSC |
| *atp*F/*atp*H | 472 | 5 | 0.010593220 | LSC |
| *psb*K/*psb*I | 386 | 4 | 0.010362694 | LSC |
| *rrn*23/rrn4.5 | 98 | 1 | 0.010204082 | IRB |
| *rrn4.5/rrn23* | 98 | 1 | 0.010204082 | IRA |
| *rpl*16 intron | 981 | 10 | 0.010193680 | LSC |
| *trn*D-GUC*/trn*Y-GUA | 403 | 4 | 0.009925558 | LSC |
| *rps*16 intron | 753 | 7 | 0.009296149 | LSC |
| *trn*R-UCU/*atp*A | 110 | 1 | 0.009090909 | LSC |
| *rps*16*/trn*Q-UUG | 1,111 | 10 | 0.009000900 | LSC |
| *rbc*L*/acc*D | 778 | 7 | 0.008997429 | LSC |
| *psb*M*/trn*D-GUC | 1,024 | 9 | 0.008789063 | LSC |
| *cem*A*/pet*A | 231 | 2 | 0.008658009 | LSC |
| *rpl*20*/clp*P | 1,047 | 9 | 0.008595989 | LSC |
| *trn*R-ACG/rrn5 | 245 | 2 | 0.008163265 | IRA |
| *rrn*5/trnR-ACG | 245 | 2 | 0.008163265 | IRB |
| *psa*I*/ycf*4 | 374 | 3 | 0.00802139 | LSC |
| *pet*A*/psb*J | 1,134 | 9 | 0.007936508 | LSC |
| *pet*N*/psb*M | 678 | 5 | 0.007374631 | LSC |
| *trn*A-UGC */rrn*23 | 143 | 1 | 0.006993007 | IRB |
| *rrn*23*/trn*A-UGC | 143 | 1 | 0.006993007 | IRA |
| *rrn*16*/trn*I-GAU | 289 | 2 | 0.006920415 | IRB |
| *trn*I-GAU*/rrn*16 | 289 | 2 | 0.006920415 | IRA |
| *trn*E-UUC*/trn*T-GGU | 598 | 4 | 0.006688963 | LSC |
| *rpo*C2*/rpo*C1 | 154 | 1 | 0.006493506 | LSC |
| *pet*B*/pet*D | 172 | 1 | 0.005813953 | LSC |
| *trn*T-UGU*/trn*L-UAA | 693 | 4 | 0.005772006 | LSC |
| *pet*D*/rpo*A | 179 | 1 | 0.005586592 | LSC |
| *pet*D intron | 753 | 4 | 0.005312085 | LSC |
| *trn*M-CAU*/atp*E | 191 | 1 | 0.005235602 | LSC |
| *ndh*B intron | 695 | 3 | 0.004316547 | IRB |
| *ndh*B intron | 695 | 3 | 0.004316547 | IRA |
| *psb*E*/pet*L | 944 | 4 | 0.004237288 | LSC |
| *rps*12 intron | 541 | 2 | 0.003696858 | IRB |
| *rps*12 intron | 541 | 2 | 0.003696858 | IRA |
| *rpl*2*/psb*A | 307 | 1 | 0.003257329 | LSC |
| *rps*12*/rrn*16 | 2,183 | 7 | 0.003206596 | IRB |
| *rrn*16*/rps*12 | 2,183 | 7 | 0.003206596 | IRA |
| *rps*4*/trn*T-UGU | 320 | 1 | 0.003125000 | LSC |
| *trn*G-UCC intron | 674 | 2 | 0.002967359 | LSC |
| *rpo*C1 intron | 706 | 2 | 0.002832861 | LSC |
| *trn*P-UGG*/psa*J | 373 | 1 | 0.002680965 | LSC |
| *trn*I-GAU intron | 935 | 2 | 0.002139037 | IRB |
| *trn*I-GAU intron | 935 | 2 | 0.002139037 | IRA |
| *trn*N-GUU*/trn*R-ACG | 562 | 1 | 0.001779359 | IRA |
| *trn*R-ACG*/trn*N-GUU | 562 | 1 | 0.001779359 | IRB |
| *ndh*B*/trn*L-CAA | 571 | 1 | 0.001751314 | IRA |
| *trn*L-CAA*/ndh*B | 571 | 1 | 0.001751313 | IRB |
| *trn*L-CAA*/ycf*15 | 673 | 1 | 0.001485884 | IRA |
| *ycf*15*/trn*L-CAA | 673 | 1 | 0.001485884 | IRB |
| *trn*A-UGC intron | 801 | 1 | 0.001248440 | IRA |
| *trn*A-UGC intron | 801 | 1 | 0.001248440 | IRB |
